# The Bacterial Markers of Identification of Invasive CovR/CovS-Inactivated Group A *Streptococcus*

**DOI:** 10.1128/spectrum.02033-22

**Published:** 2022-10-06

**Authors:** Yong-An Shi, Tzu-Ching Chen, Yan-Wen Chen, Yen-Shan Liu, Yi-Ywan M. Chen, Chih-Ho Lai, Cheng-Hsun Chiu, Chuan Chiang-Ni

**Affiliations:** a Graduate Institute of Biomedical Sciences, College of Medicine, Chang Gung University, Taoyuan, Taiwan; b Department of Microbiology and Immunology, College of Medicine, Chang Gung University, Taoyuan, Taiwan; c Molecular Infectious Disease Research Center, Chang Gung Memorial Hospital at Linkou, Taoyuan, Taiwan; d Department of Orthopedic Surgery, Chang Gung Memorial Hospital at Linkou, Taoyuan, Taiwan; e Department of Medical Biotechnology and Laboratory Science, College of Medicine, Chang Gung University, Taoyuan, Taiwan; Griffith University

**Keywords:** CovR/CovS, PepO, RocA, SpeB, SIP, group A *Streptococcus*

## Abstract

Necrotizing fasciitis is a severe infectious disease that results in significant mortality. Streptococcus pyogenes (group A Streptococcus, GAS) is one of the most common bacterial pathogens of monomicrobial necrotizing fasciitis. The early diagnosis of necrotizing fasciitis is crucial; however, the typical cutaneous manifestations are not always presented in patients with GAS necrotizing fasciitis, which would lead to miss- or delayed diagnosis. GAS with spontaneous inactivating mutations in the CovR/CovS two-component regulatory system is significantly associated with destructive diseases such as necrotizing fasciitis and toxic shock syndrome; however, no specific marker has been used to identify these invasive clinical isolates. This study evaluated the sensitivity and specificity of using CovR/CovS-controlled phenotypes to identify CovR/CovS-inactivated isolates. Results showed that the increase of hyaluronic acid capsule production and streptolysin O expression were not consistently presented in CovS-inactivated clinical isolates. The repression of SpeB is the phenotype with 100% sensitivity of identifying in CovS-inactivated isolates among 61 clinical isolates. Nonetheless, this phenotype failed to distinguish RopB-inactivated isolates from CovS-inactivated isolates and cannot be utilized to identify CovR-inactivated mutant and RocA (**R**egulator **o**f **C**ov)-inactivated isolates. In this study, we identified and verified that PepO, the endopeptidase which regulates SpeB expression through degrading SpeB-inducing quorum-sensing peptide, was a bacterial marker to identify isolates with defects in the CovR/CovS pathway. These results also inform the potential strategy of developing rapid detection methods to identify invasive GAS variants during infection.

**IMPORTANCE** Necrotizing fasciitis is rapidly progressive and life-threatening; if the initial diagnosis is delayed, deep soft tissue infection can progress to massive tissue destruction and toxic shock syndrome. Group A Streptococcus (GAS) with inactivated mutations in the CovR/CovS two-component regulatory system are related to necrotizing fasciitis and toxic shock syndrome; however, no bacterial marker is available to identify these invasive clinical isolates. Inactivation of CovR/CovS resulted in the increased expression of endopeptidase PepO. Our study showed that the upregulation of PepO mediates a decrease in SpeB-inducing peptide (SIP) in the *covR* mutant, indicating that CovR/CovS modulates SIP-dependent quorum-sensing activity through PepO. Importantly, the sensitivity and specificity of utilizing PepO to identify clinical isolates with defects in the CovR/CovS pathway, including its upstream RocA regulator, were 100%. Our results suggest that identification of invasive GAS by PepO may be a strategy for preventing severe manifestation or poor prognosis after GAS infection.

## INTRODUCTION

Necrotizing fasciitis is a bacterial infectious disease with mortality exceeding 70%, characterized by rapidly progressive soft tissue necrosis, sepsis, and toxic shock syndrome (1). Among Gram-positive bacteria, Streptococcus pyogenes (group A Streptococcus, GAS) is the leading pathogen causing monomicrobial necrotizing fasciitis (1). Cutaneous manifestations are not always initially present in patients with GAS necrotizing fasciitis (1, 2). Therefore, the infection is often misdiagnosed or the correct diagnosis is delayed (1, 3), which could result in severe acute complications in patients. Developing predictive markers for identifying high-risk patients is crucial for preventing and diagnosing necrotizing fasciitis. Currently, early diagnosis of necrotizing fasciitis is dependent on the observation of manifestations in the clinic and the detection of surrogate markers (e.g., The Laboratory Risk Indicator for Necrotizing Fasciitis [LRINEC] scoring system) in the laboratory (1, 4). Nonetheless, whether the LRINEC scoring system is useful for the early recognition of GAS necrotizing fasciitis is still under debate (5–8). Also, no bacterial biomarker is available for the early diagnosis of GAS necrotizing fasciitis.

GAS isolates are classified by the widely used single-locus sequence *emm-*typing system. More than 250 different *emm* types have been identified, with *emm*1- and *emm*3-GAS frequently associated with severe clinical manifestations (9–11). Furthermore, spontaneous inactivating mutations in genes encoding the CovR/CovS (**c**ontrol **o**f **v**irulence) two-component regulatory system are significantly related to necrotizing fasciitis and toxic shock syndrome (12–14). CovR is phosphorylated by CovS and the phosphorylated response CovR protein mediates the transcriptional repression of virulence factors including streptolysin S (SLS), streptolysin O (SLO), streptokinase, DNase Sda1, hyaluronic acid capsule, M protein, and C5a peptidase (15–19). The spontaneous CovR/CovS-inactivated mutations occur during GAS infection; the derepression of virulence factors expression in both *covS* and *covR* mutants contributes to increased resistance to immune clearance, enhances invasion activity, and may increase the risk of systemic infection (14, 18, 20, 21). Intriguingly, the phenotypes of the *covS*-deletion and *covR*-deletion mutants are not identical. The expression of SpeB cysteine protease, DNase Spd3, and protein G-related α2 M-binding protein (Grab) is upregulated in the *covR*-deletion mutant but downregulated in the *covS*-deletion mutant (14, 20–22). In this study, therefore, we referred to the *covS*-deletion, CovS-truncation, and CovS kinase-inactivated isolates as the CovS-inactivated isolates (expressing nonphosphorylated CovR) and to the *covR*-deletion and CovR-truncation isolates as the CovR-inactivated isolates (have no CovR protein). The phosphorylation of CovR is also modulated by RocA (**r**egulator **o**f **C**ov) in a CovS-dependent manner (23–26). Previous studies have demonstrated that *rocA* mutants show decreased levels of CovR phosphorylation and increased bacterial virulence (26–28). Notably, *emm*3 isolates have a truncated RocA; this truncation is responsible for increased capsule expression and may contribute to the association of *emm*3 GAS with severe manifestations (29, 30).

The *emm* typing and identification of *rocA* truncation or CovR/CovS spontaneous mutations by Sanger sequencing are time- and labor-consuming. The increased hyaluronic acid capsule expression in the *covR/covS* mutants would result in an encapsulated or mucoid colony morphology; however, we found that 69% (20/29) of CovS-inactivated clinical isolates did not have a distinguishable encapsulated colony morphology, indicating that colony morphology is not a reliable characteristic of CovS-inactivated isolates. The early diagnosis of necrotizing fasciitis is critically important. As an alternative indicator of infection severity (e.g., the LRINEC scoring system), this study suggested that the bacterial marker PepO endopeptidase could be utilized to monitor the appearance of CovR/CovS- and RocA-inactivated mutants during GAS infection. Also, this finding reveals a novel strategy, identification of bacterial markers of CovR/CovS-inactivated mutants during infection, for the prevention of invasive GAS infection.

## RESULTS

### Encapsulated colony morphology is not a reliable phenotype for the identification of CovS-inactivated clinical isolates.

**FIG 1:**
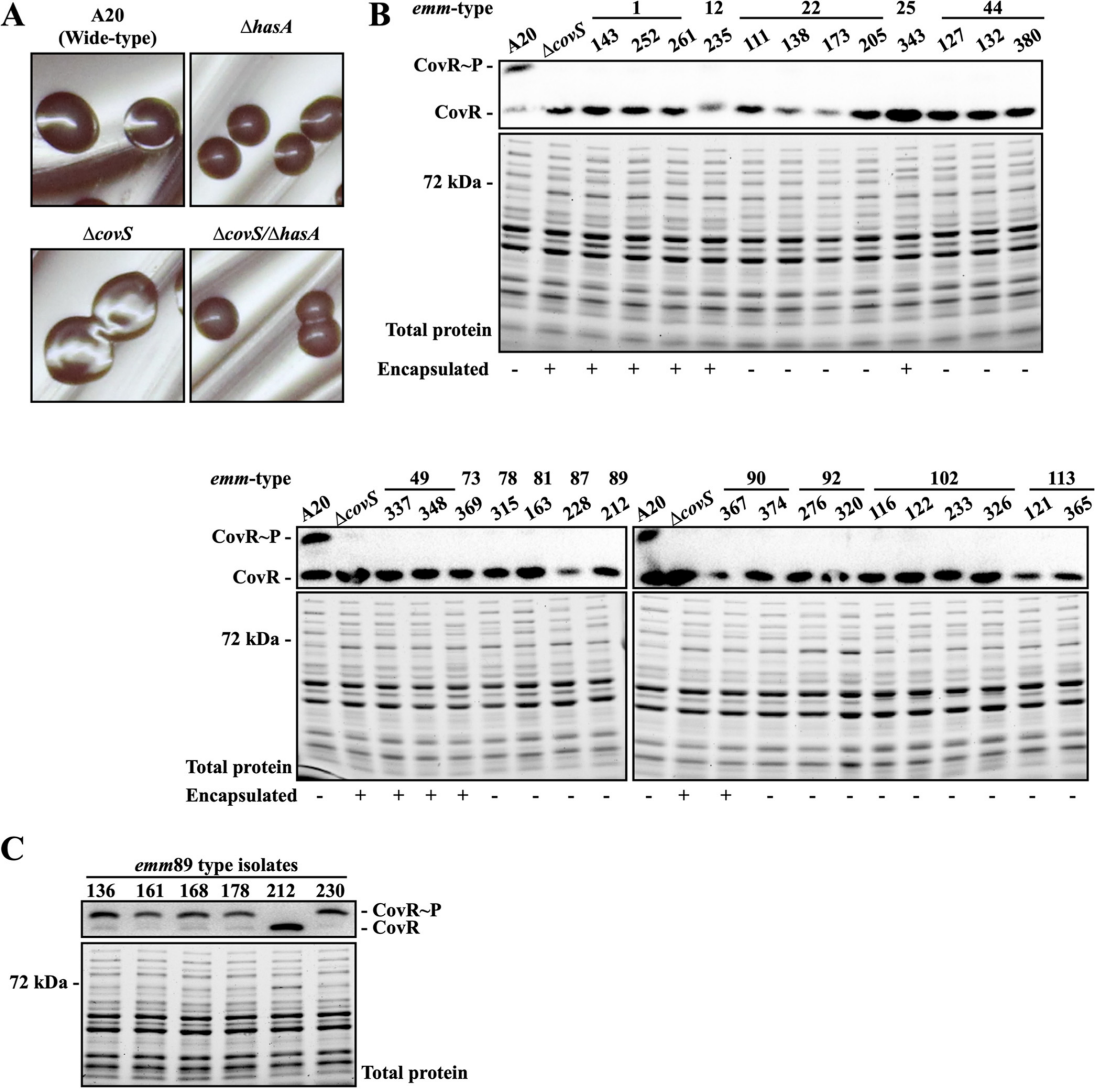
Colony morphology and expression of phosphorylated CovR in the selected clinical isolates and in the wild-type A20 strain and its *covS* isogenic mutants. (A) Colony morphology of the wild-type A20 strain, the *covS* mutant, and their *hasA* mutants. (B) Expression of phosphorylated CovR and the total protein profile upon SDS-PAGE of selected clinical isolates. (C) Phosphorylation level of CovR in the *emm*89 isolates. The phosphorylated CovR protein was detected by Phostag Western blotting. Encapsulated colonies are denoted by a plus (+) and unencapsulated colonies are denoted by a minus (−) as shown below the SDS-PAGE gel and Western blot images.

CovR/CovS regulates hyaluronic acid capsule synthesis by inhibiting transcription of the *hasABC* operon (16, 19); therefore, an encapsulated (or mucoid) colony morphology is the typical phenotype of *covR* and *covS* mutants (Fig. 1A). Our previous study identified, by Phostag Western blotting (Fig. 1B), 29 CovS-inactivated clinical isolates (31). In this study, we found that only nine isolates (31%) exhibited a distinguishable encapsulated colony morphology (Fig. 1B and data not shown). *hasA* and *hasB*, but not *hasC*, are required for the hyaluronic acid capsule synthesis (32). Therefore, the *hasA* and *hasB* sequences of the 20 CovS-inactivated isolates with a normal colony morphology were analyzed, and the results showed that SPY163 (*emm*81), SPY228 (*emm*87), SPY173 (*emm*22), and SPY121 (*emm*113) had frameshift mutations in *hasA* or *hasB* (Table 1). In addition, the *emm*89-type isolate (SPY212) had no *hasABC* operon (data not shown). Due to the lack of a *hasABC* operon, the colony morphology of the CovS-inactivated SPY212 was not distinguishable from those of other *emm*89 isolates (Fig. 1C and Fig. S1). The remaining 15 CovS-inactivated isolates possessed no deletion or frameshift mutations in *hasA* and *hasB*. The *emm*22, *emm*102, and *emm*113 isolates had missense mutations in *hasA* and *hasB* (Table 1); however, whether these amino acid replacements are involved in inactivating HasA and HasB activity is unknown. These results indicated that in isolates with or without the functional *hasABC* operon, an encapsulated colony morphology is not a reliable phenotype for identifying CovS-inactivated isolates.

**TABLE 1:** Nucleotide sequence of *hasA* and *hasB* in the phosphorylated CovR-negative and non-encapsulated GAS isolates^*a*^

Isolate no.	*emm* type	*hasA*	*hasB*
SPY111	22		S109N, D402V
SPY138	22		S109N
SPY173	22		S109N; c394t = stop at aa 132
SPY205	22		S109N
SPY127	44		
SPY132	44		
SPY380	44		
SPY315	78		
SPY163	81	g224t = stop at aa 82	
SPY228	87	1 bp insert = stop at aa 47	
SPY212	89	Absent	Absent
SPY374	90		
SPY276	92		
SPY320	92		
SPY116	102	I41V	I235V, R305Q, N400G
SPY122	102	I41V	I235V, R305Q, N400G
SPY233	102	I41V	I235V, R305Q, N400G
SPY326	102	I41V	I235V, R305Q, N400G
SPY121	113		Y52S; 1 bp delete = stop at aa 105
SPY365	113	Y232D	Y52S

a aa, amino acid. Reference sequences (NCBI accession no.): *emm*22: NZ_CP035438.1, NZ_CAAIOC010000006.1, NZ_CAAJES010000006.1, NZ_CAAIJZ010000003.1; *emm*44: NZ_CAAIYK010000002.1, NZ_CAAIXB010000002.1; *emm*78: CP035437.1, NZ_CAAJDX010000002.1; *emm*81: CP027771.1, NZ_CAAJCN010000008.1; *emm*87: NZ_CAAIZD010000010.1, NZ_CAAIUZ010000009.1; *emm*90: CP035444.1, NZ_CAAJFV010000002.1; *emm*102: NZ_CAAIOC010000006.1, NZ_CAAHKE010000006.1, NZ_CAAJES010000006.1; *emm*113: NZ_CAAIYN010000002.1, NZ_CAAIXY010000002.1, NZ_CAAIXF010000003.1, CAAHKQ010000017.1.

### SpeB expression level is the negative predictive marker of CovS-inactivated isolates.

**FIG 2:**
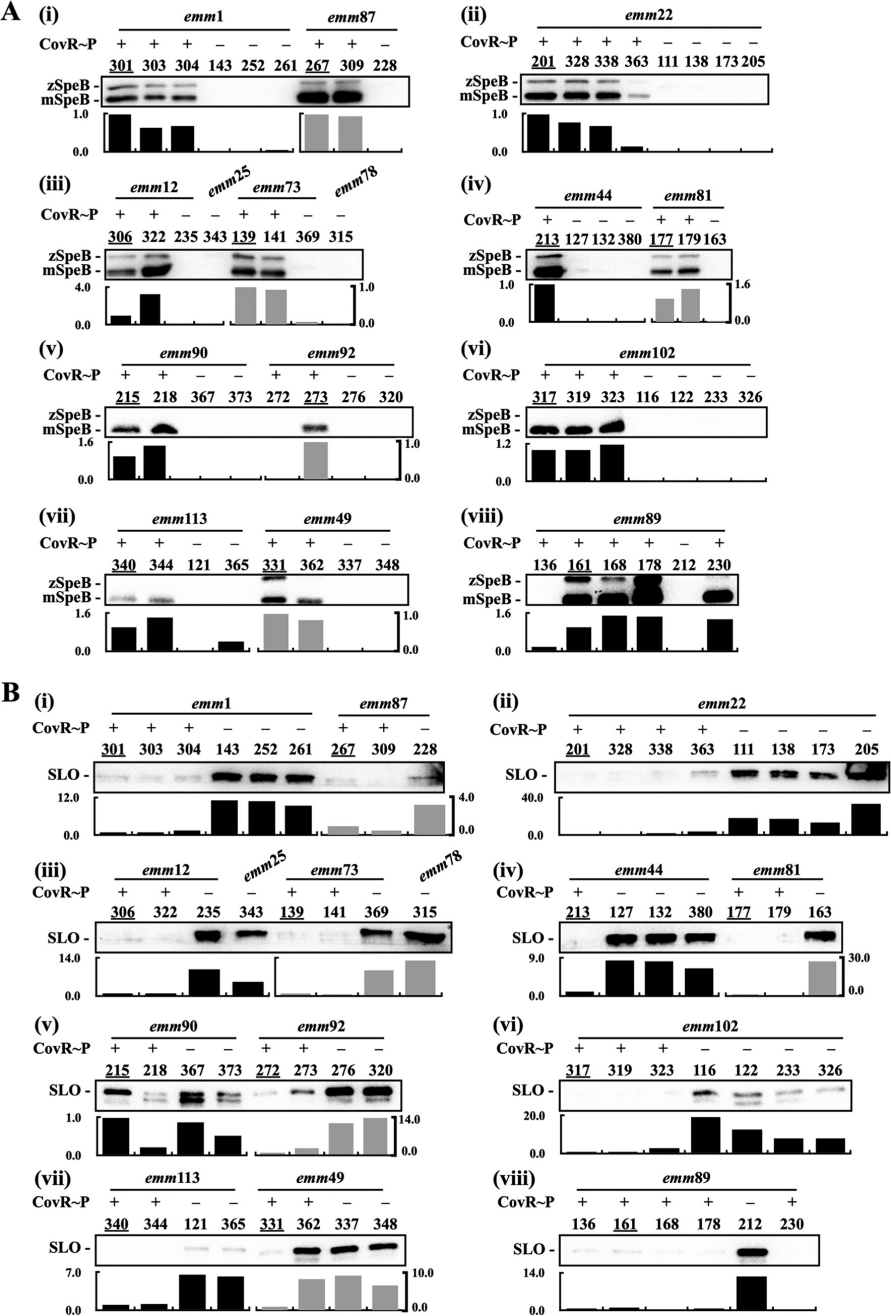
Expression of SpeB and SLO in the CovR/CovS-activated and CovS-inactivated isolates. Group A *Streptococcus* (GAS) clinical isolates were grown to an optical density at 600 nm (OD_600_) of 1.0 and the bacterial culture supernatants were collected for analysis. Expression of SpeB (A) and SLO (B) in the CovR/CovS-activated and CovS-inactivated isolates was analyzed by Western blot hybridization. Lower panels show relative band density (relative to the isolates labeled with an underline on each blot). CovR~P denotes phosphorylated CovR; CovR denotes non-phosphorylated CovR. zSpeB, SpeB zymogen form; mSpeB, SpeB mature form.

CovR is phosphorylated by CovS, and the expression of the cysteine protease SpeB is inhibited by the phosphorylated CovR (17, 33). The expression of SpeB in the *covR*-deletion mutant is increased compared to that in the wild-type strain; however, in the *covS* mutant, in which CovR cannot be phosphorylated, SpeB expression is repressed (14, 20–22). Therefore, the repression of SpeB is the phenotype which has been utilized for screening CovS-inactivated mutants in mouse infection models (18, 34, 35). To evaluate whether the SpeB expression could be utilized as the predictive marker for the CovS-inactivated isolates, the 61 CovR/CovS-activated and CovS-inactivated clinical isolates were included, and the supernatant from these isolates was collected for detecting SpeB cysteine protease by Western blot hybridization. The expression of SpeB, including the zymogen and mature forms, was detected in all isolates which expressed phosphorylated CovR except for SPY272 (*emm*92) and SPY136 (*emm*89) (Fig. 2A, subpanels v and viii). All CovS-inactivated isolates showed no SpeB expression (Fig. 2A). Based on these results, the positive and negative predictive values for detecting CovS-inactivated isolates by SpeB are 0.94 and 1.0, respectively. The sensitivity and specificity are 100% and 93.75%, respectively (Table 2 and Table S1 in the supplemental material).

**TABLE 2:** Positive predictive value, negative predictive value, sensitivity, and specificity of SpeB, SLO, and PepO as markers for identifying CovS-inactivated isolates^*a*^

Marker and no. of isolates	TP	FP	TN	FN	PPV	NPV	Sensitivity (%)	Specificity (%)
SpeB	CovR~P (−)/SpeB (−)	CovR~P (+)/SpeB (−)	CovR~P (+)/SpeB (+)	CovR~P (−)/SpeB (+)	TP/(TP+FP)	TN/(TN+FN)	TP/(TP+FN)	TN/(FP+TN)
Isolates (*n*)	29	2	30	0	0.94	1.0	100	93.75
SLO	CovR~P (−)/SLO (++)	CovR~P (+)/SLO (++)	CovR~P (+)/SLO (−/+)	CovR~P (−)/SLO (−/+)				
Isolates (*n*)	21	2	30	8	0.91	0.79	72.41	93.25
PepO	CovR~P (−)/PepO (++)	CovR~P (+)/PepO (++)^*b*^	CovR~P (+)/PepO (−/+)	CovR~P (−)/PepO (−/+)				
Isolates (*n*)	29	1	31	0	0.97	1.0	100	96.88

a TP, true positive; FP, false positive; TN, true negative; FN, false negative; PPV, positive predictive value; NPV, negative predictive value.

b RocA-inactivated isolate (SPY362).

The expression of streptolysin O is inhibited by both phosphorylated and nonphosphorylated CovR (21); therefore, the expression of SLO is upregulated in CovR- and CovS-inactivated mutants. The anti-SLO antibody is commercially available and the SLO expression level was utilized in this study for identifying CovS-inactivated isolates. Higher levels of SLO expression were generally observed in the CovS-inactivated isolates compared to the CovS-activated isolates (except SPY215, see Fig. 2B panel v); however, a strong, clear SLO signal was not revealed in some of the CovS-inactivated isolates, including SPY228 (*emm*87, Fig. 2B subpanel i), SPY373 (*emm*90, subpanel v), SPY223, SPY326 (*emm*102, subpanel vi), SPY121, and SPY365 (*emm*113, subpanel vii). Therefore, the positive and negative predictive values for detecting CovS-inactivated isolates by SLO are 0.91 and 0.79, respectively. The specificity is 93.25%; however, the sensitivity is only 72.41% (Table 2 and Table S1). Notably, it was difficult to evaluate SLO expression levels in some GAS isolates because SLO expression levels were not equally similar among different *emm* type isolates [e.g., the *emm*90, *emm*92, and *emm*102 isolates; Fig. 2B subpanels v and vi).

### Identification of an overexpressed 72-kDa protein in the *covR* and *covS* mutants.

**FIG 3:**
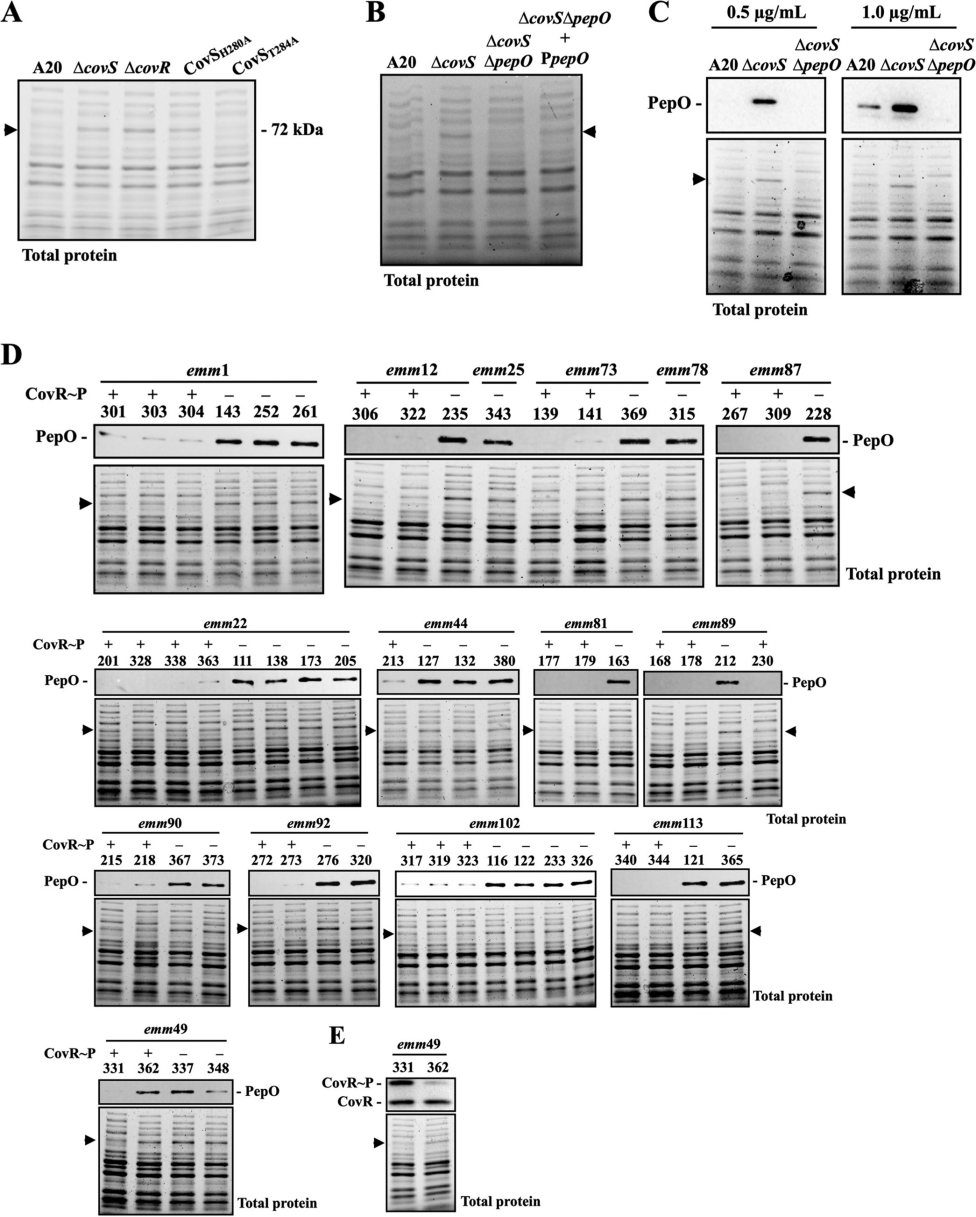
Identification and verification of the 70- to 72-kDa protein marker in the *covR/covS* mutants and the CovR/CovS-activated and -inactivated isolates. (A) Total protein profiles of the wild-type A20 strain, *covS* mutant (Δ*covS*), *covR* mutant (Δ*covR*), CovS kinase-inactivated mutant (CovS_H280A_), and CovS phosphatase-inactivated mutant (CovS_T284A_). (B) Total protein profiles of A20, *covS* mutant (Δ*covS*), *covR* mutant (Δ*covR*), *covS*/*pepO* double mutant (Δ*covS*/Δ*pepO*), and *pepO trans*-complementary strain (Δ*covS*/Δ*pepO*+P*pepO*). (C) Western blot of PepO expression in A20, *covS* mutant (Δ*covS*), and *covS*/*pepO* double mutant (Δ*covS*/Δ*pepO*) detected by the anti-PDTTYYEEGNEKAEELR antibody. (D) Comparison of PepO expression in CovR/CovS-activated and CovS-inactivated clinical isolates. The PepO protein was detected by the anti-PDTTYYEEGNEKAEELR antibody in a total protein extract. (E) Phosphorylation levels of CovR in the *emm*49 SPY331 and SPY362 isolates. CovR~P denotes phosphorylated CovR; CovR denotes non-phosphorylated CovR. Arrows indicate when a signal from only the CovR/CovS-inactivated mutants was identified. The lower sections of the image in panels C, D, and E show total protein as the loading control.

In this study, we identified an unknown protein (about 70 to 72 kDa) which can be clearly visualized on 10% SDS-PAGE of total proteins from all CovS-inactivated isolates (Fig. 1B) and the *covS* mutant, *covR* mutant, and CovS kinase-inactivated mutant (CovS_H280A_) (Fig. 3A); however, this unknown protein was not detected in total proteins from the wild-type A20 strain or its CovS phosphatase-inactivated mutant (CovS_T284A_) (Fig. 3A). These findings suggest that this unknown protein could be a potential marker for identifying both CovR- and CovS-inactivated isolates. Protein identification by mass spectrometry analysis showed that the three most abundant proteins were endopeptidase PepO (72 kDa), proline tRNA ligase (69 kDa), and oligoendopeptidase F (69 kDa). To confirm the identity of the unknown protein, a Δ*covS*/Δ*pepO* mutant was constructed and the total protein of the wild-type strain, *covR* mutant, *covS* mutant, and Δ*covS*/Δ*pepO* mutant were extracted and analyzed by SDS-PAGE. The results showed that the 70- to 72-kDa protein signal was not observed in the wild-type strain nor in the Δ*covS*/Δ*pepO* mutant (Fig. 3B). Furthermore, the expression of this 70- to 72-kDa protein was restored in the *pepO trans*-complementary strain (Fig. 3B), indicating that this previously unidentified protein is PepO.

Western blot hybridization analysis was performed to confirm the identity of the 70- to 72-kDa protein as PepO. In brief, we produced a polyclonal antibody against the synthetic predicted antigenic PepO peptide (PDTTYYEEGNEKAEELR) to detect the protein. As a negative control, we observed no signal in the 70- to 72-kDa range for the Δ*covS*/Δ*pepO* mutant (Fig. 3C). Furthermore, PepO expression was upregulated in the *covR* and *covS* mutants compared to that in the wild-type A20 strain from the log to the stationary growth phases (Fig. S2). These results confirm that the overexpressed 70- to 72-kDa protein in the CovR*/*CovS-inactivated mutants is PepO. Additionally, Western blotting with the anti-PepO antibody (0.1 μg/mL) was used to investigate whether PepO could be used as a marker to identify CovS-inactivated clinical isolates. As shown in Fig. 3D, with only one exception, SPY362 (*emm*49), the CovS-inactivated isolates showed a stronger PepO signal than the CovR/CovS-activated isolates. SPY362 showed a weak phosphorylated CovR signal but expressed PepO at a level comparable to that of the CovS-inactivated *emm*49 isolates (SPY337 and SPY348; Fig. 3D and E). Genome sequencing analysis (using the MiSeq system) of SPY362 showed single-nucleotide polymorphisms (SNPs) in CovR/CovS, HasA, HasC, and SpeB compared to the *emm*49 reference NZ131 strain (NCBI accession no. CP000829.1; Table S2). The amino acid sequence of CovR/CovS in SPY362 was identical to that of the *emm*1-type A20 strain (NCBI accession no. CP003901.1) except for a common SNP (I332V) found in CovS, suggesting that CovR/CovS should be functional in SPY362. Nonetheless, the deletion of a single unit of the GAAGGA variable-number tandem-repeat (VNTR) in the *rocA* promoter region was identified by Sanger sequencing. Based on these results, the positive and negative predictive values for identifying CovR/CovS-inactivated isolates by PepO are 0.97 and 1.0, respectively. The sensitivity and specificity are 100% and 96.88%, respectively (Table 2 and Table S1).

### PepO could be utilized to identify RocA-inactivated isolates and *emm*3-type isolates.

**FIG 4:**
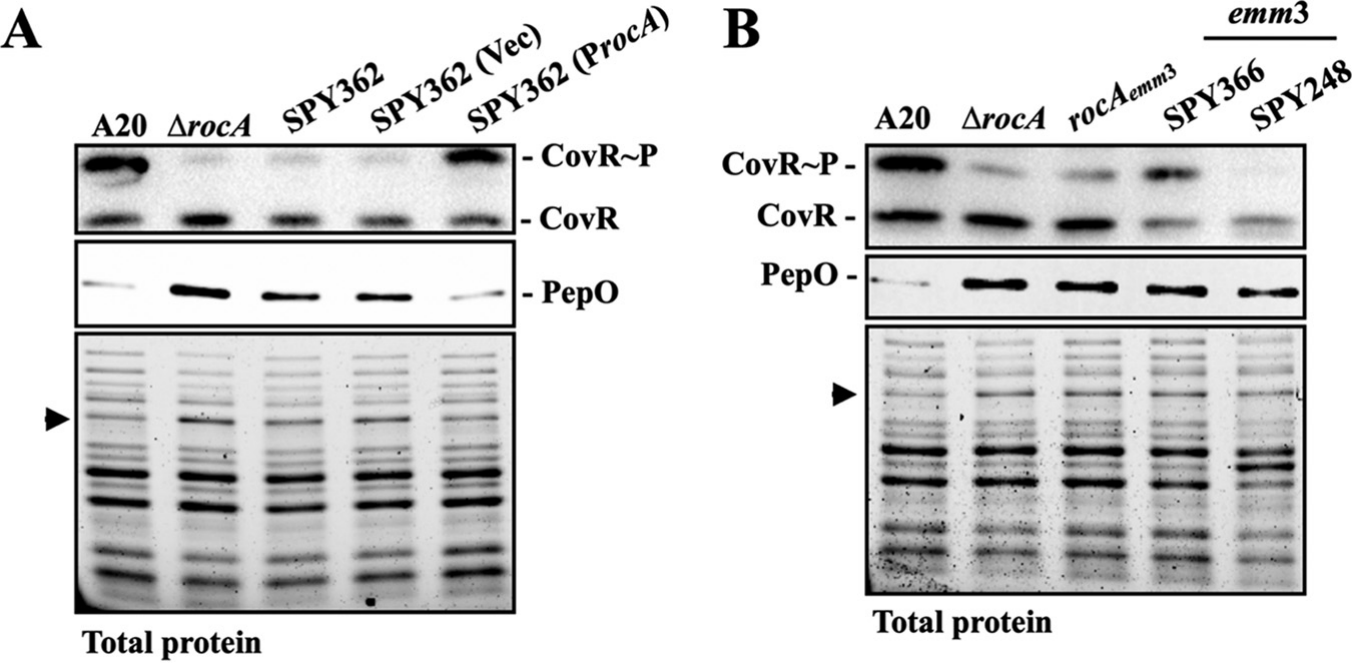
Expression levels of PepO and phosphorylated CovR in the wild-type strains, the *rocA* mutants, the *rocA trans*-complementary strain, and the *emm*3-type clinical isolates. (A) The expression level of PepO and phosphorylated CovR in the *emm*1 wild-type A20 strain, its *rocA* mutant (Δ*rocA*), SPY362 (*emm*49), and its vector-control (Vec), and the *rocA trans*-complementary strain (P*rocA*). (B) Expression levels of PepO and phosphorylated CovR in the *emm*1 wild-type A20 strain, its *rocA* mutant (Δ*rocA*), the RocA-truncated mutant (*rocA_emm_*_3_), and the *emm*3-type isolates. PepO and phosphorylated CovR were detected by Western blotting and Phostag Western blotting, respectively, from a total protein extract. CovR~P denotes phosphorylated CovR; CovR denotes non-phosphorylated CovR. Lower panels of images show total protein as the internal loading control. An arrow indicates the PepO signal on SDS-PAGE.

RocA is an accessory protein that modulates CovR phosphorylation through CovS (23–26, 36). Zhu et al. (28) showed that deletion of a single unit of the GAAGGA in the *rocA* promoter region results in the downregulation of CovR phosphorylation. To determine whether the decrease in CovR phosphorylation in the SPY362 isolate was mediated by RocA, the wild-type *rocA* gene from the *emm*1-type A20 strain was *trans*-complemented into SPY362, and the expression of PepO and phosphorylated CovR in the complementary strain was analyzed. As the experimental control, the *rocA* isogenic mutant showed a decrease in phosphorylated CovR expression compared to the wild-type A20 strain (Fig. 4A). Complementation of the *rocA* gene from the *emm*1-type A20 strain into SPY362 upregulated the expression of phosphorylated CovR in comparison with SPY362 and its vector-control strain (Fig. 4A). Expression of PepO was increased in the A20 *rocA* isogenic mutant compared with that in the wild-type A20 strain; furthermore, the expression of PepO was decreased in the *rocA trans*-complementary strain to a level similar to that in A20 (Fig. 4A). These results indicate that the upregulation of PepO expression in SPY362 is mediated by RocA. Also, these results indicated that the repression of SpeB cannot be utilized to detect a RocA-inactivated isolate (SPY362, Fig. 2A panel vii). After including the RocA-inactivated isolate, the positive predictive value for detecting CovS-inactivated clinical isolates by PepO increased from 0.97 to 1.0 and the specificity increased from 96.88% to 100%.

Studies have shown that *emm*3-type isolates have a truncated RocA and express lower levels of phosphorylated CovR than *emm*1-type isolates (29, 30). Therefore, we further verified whether PepO could be used to identify *emm*3 isolates. First, we analyzed whether the expression of PepO was upregulated in the RocA-truncated mutant. To achieve this, the *rocA* gene of the *emm*1 A20 strain was replaced by the *rocA* gene from the *emm*3 isolate (SPY248), producing a RocA-truncated mutant (*rocA_emm_*_3_, Fig. 4B) (26). Next, we used Western blot hybridization to determine whether PepO could be a marker for identifying the RocA-truncated mutant and *emm*3 isolates. Phostag Western blot analysis showed the presence of phosphorylated CovR in the A20 *rocA* isogenic mutants, the RocA-truncated mutant, and SPY366 (*emm*3), but not in SPY248 (*emm*3) (Fig. 4B). The expression of PepO was significantly upregulated in the *rocA* mutants and *emm*3 isolates compared to that in the wild-type A20 strain (Fig. 4B). Also, the PepO signal in *rocA* mutants and *emm*3 isolates was also visualized by SDS-PAGE (Fig. 4B). These results indicate that PepO can also be used to identify *emm*3 isolates.

### PepO mediates the decrease of SIP-inducing *speB* expression in the *covR* mutant.

**FIG 5:**
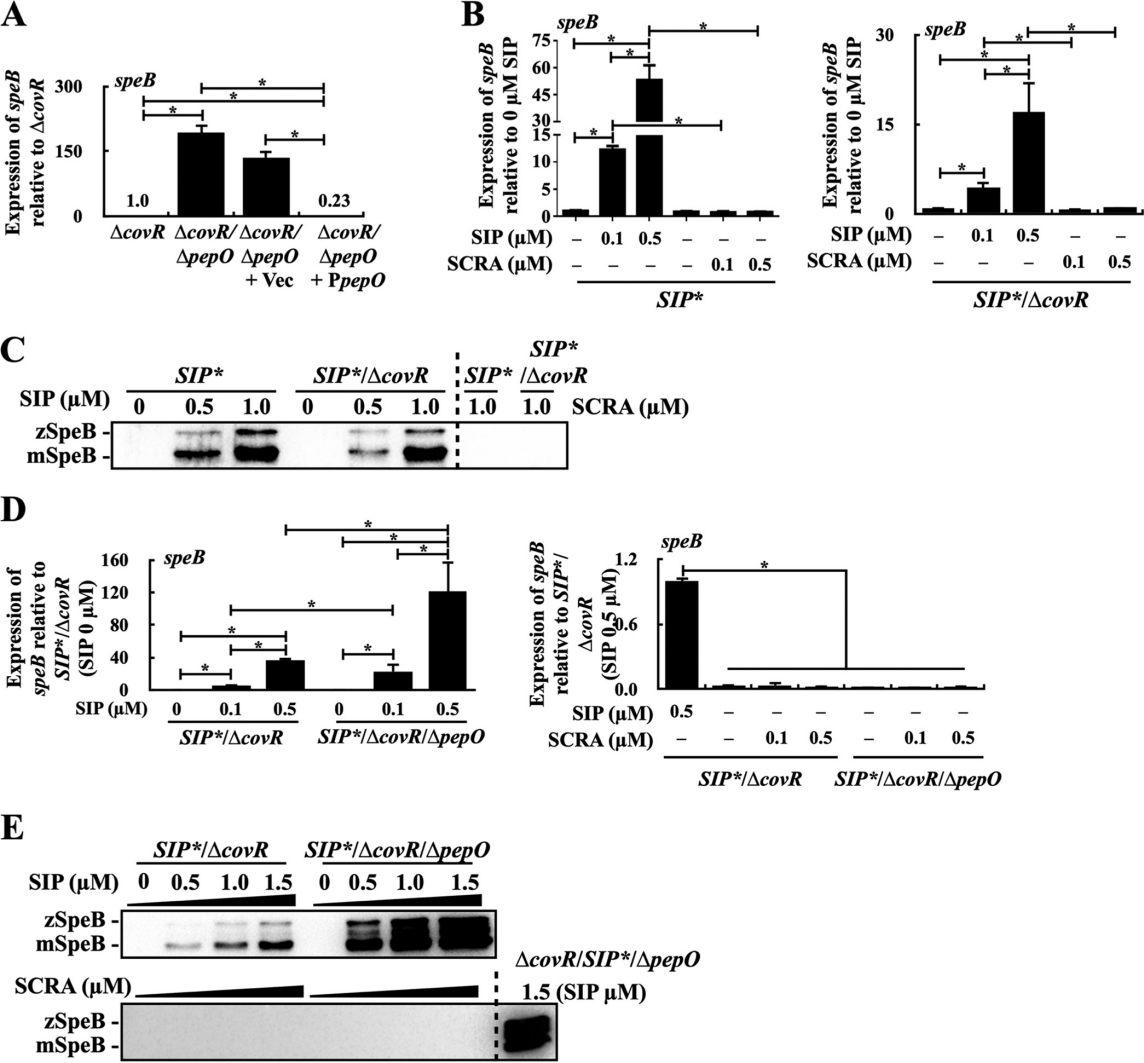
Expression of SpeB in the *covR* mutant and its Δ*pepO* and SIP-inactivated (SIP*) mutants. (A) Expression of *speB* in the Δ*covR* mutant, Δ*covR*/Δ*pepO* mutant, the vector-control (Vec) strain, and the *pepO trans*-complementary (P*pepO*) strain. (B and C) Transcription of (B) *speB* and (C) expression of SpeB in the SIP* and SIP*/Δ*covR* mutants in the treatment with the synthetic SIP peptide and scramble peptide (SCRA). (D and E) The expression of (D) *speB* and (E) SpeB in the SIP*/Δ*covR* and SIP*/Δ*covR*/Δ*pepO* mutants in the treatment with synthetic SIP and SCRA peptides. GAS strains were grown to OD_600_ = 0.8 in tryptic soy broth supplemented with yeast (TSBY broth), and bacterial pellets were collected and incubated in acidic TSBY broth (pH 6.0) supplemented with different concentrations of SIP and SCRA for 1 h. Culture supernatant was used for detecting SpeB. zSpeB, zymogen form of SpeB; mSpeB, mature form of SpeB. Bacterial RNA was extracted for real-time quantitative PCR analysis. The expression of *speB* was normalized to that of *gyrA*. *, *P < *0.05.

Brouwer et al. (37) showed that the *speB* expression is negatively regulated by PepO. The expression of PepO was upregulated in the CovR/CovS- and RocA-inactivated isolates (Fig. 3 and 4); however, the repression of *speB* is found in the *covS* mutant but not in the *covR* mutant (14, 20–22, 27). Therefore, the present study further elucidated the role of PepO in regulating SpeB expression in the *covR* mutant. We constructed a Δ*covR*/Δ*pepO* mutant and compared *speB* expression in the *covR* mutant (Δ*covR*) to that in the Δ*covR*/Δ*pepO* mutant. The results showed that the deletion of *pepO* in the *covR* mutant upregulated *speB* expression (Fig. 5A). In addition, the *trans*-complementation of *pepO* in the Δ*covR*/Δ*pepO* mutant repressed *speB* expression (Fig. 5A). These results indicated that PepO acts as the repressor of *speB* transcription in the *covR* mutant.

*speB* expression is activated by the quorum-sensing RopB regulator with binding to the SpeB-inducing peptide (38–40). PepO is an endopeptidase which disrupts the RopB-independent quorum-sensing pathway (Rgg2/3) by degrading the quorum-sensing short hydrophobic peptide (41). This study, therefore, elucidated whether the upregulation of PepO in the *covR* mutant would degrade SIP to downregulate SIP-inducing SpeB expression. To exclude the signal from the endogenous SIP, SIP-inactivated mutants (SIP*) of the wild-type strain and the *covR* mutant were utilized for analyses. The results showed that supplementation with SIP but not scrambled peptide (SCRA) activated *speB* transcription in the SIP* mutant and SIP*/Δ*covR* mutant; noticeably, the 0.1- and 0.5-μM SIP treatments induced a 12- and 52-fold increases in *speB* transcription in the SIP* mutant, while 4- and 17-fold increases in the SIP*/Δ*covR* mutant were observed (Fig. 5B). In line with Fig. 5B, a Western blot analysis showed that the SIP* mutant expressed a higher level of SpeB than the SIP*/Δ*covR* mutant under 0.5-μM SIP treatments (Fig. 5C), indicating that the SIP*/*ΔcovR* mutant is less susceptible to SIP stimuli than the SIP* mutant.

To demonstrate that PepO could modulate the intracellular concentration of SIP in the *covR* mutant, a *pepO* mutant of the SIP*/*ΔcovR* mutant was constructed and SpeB expression was analyzed in the SIP*/Δ*covR* and SIP*/Δ*covR*/Δ*pepO* mutants under treatment with different concentrations of SIP. Supplementation with SIP but not SCRA during incubation activated SpeB expression at both the transcriptional and translational levels in the SIP*/Δ*covR* and SIP*/Δ*covR*/Δ*pepO* mutants (Fig. 5D and E). Under treatments with the same concentrations of SIP, quantitative PCR (qPCR) and Western blot analyses showed that the SIP*/Δ*covR*/Δ*pepO* mutant expressed a higher level of SpeB than the SIP*/Δ*covR* mutant (Fig. 5D and E), suggesting that PepO regulates *speB* expression by degrading SIP in the *covR* mutant.

## DISCUSSION

An encapsulated, or mucoid, colony morphology is one of the most identifiable phenotypes of CovR/CovS-inactivated mutants (16). However, in the present study, 69% (20/29) of CovS-inactivated isolates did not exhibit an encapsulated colony morphology. We did not determine whether the transcription of *hasA* was upregulated in these CovS-inactivated isolates; however, our results suggest that the presence of encapsulated colony morphology is not a reliable characteristic for identifying CovS-inactivated clinical isolates. Importantly, the unexpectedly low rate of encapsulated phenotype expression in CovS-inactivated GAS isolates may result in underestimation of their prevalence.

An anti-SLO antibody is commercially available, and increased SLO expression is observed in both CovR- and CovS-inactivated mutants (14, 20, 21, 42). Nonetheless, the negative predictive value and the sensitivity of identifying CovS-inactivated isolates by SLO were only 0.79 and 72.41%, respectively, indicating that SLO is not a sensitive marker for identifying these invasive isolates. The cysteine protease SpeB is the most abundantly secreted protein in GAS culture supernatant (43, 44). Although the repression of SpeB is not a phenotype for identifying CovR-inactivated mutants, this phenotype is highly sensitive to identifying CovS-inactivated isolates. Nonetheless, SpeB expression is regulated by multiple regulators, and mutations in the SpeB-positive regulator RopB could also result in repressed SpeB expression (45). Further, SpeB expression is restricted during the specific growth phase (45–47), and quantification of secreted proteins is difficult because of the lack of reliable internal control proteins in bacterial culture supernatants. These factors increased the difficulty of using SpeB as a bacterial maker for identifying CovS-inactivated isolates.

Phosphorylation of CovR is modulated by RocA in a CovS-dependent manner (24–26). There is no phosphorylation of CovR in the CovS-inactivated mutants; however, in the *rocA* mutant, CovR phosphorylation is decreased during the exponential growth phase but increases to a level similar to that in the wild-type strain during the stationary phase (26, 27). Similarly, in the *rocA* mutant, SpeB expression is repressed during the exponential growth phase but upregulated during the stationary phase compared with that in the wild-type strain (27). Therefore, although *rocA*-inactivating mutations result in the inactivation of CovR phosphorylation during the exponential phase of growth, *rocA* mutants cannot be identified by the detection of phosphorylated CovR or SpeB. The present study showed that PepO expression was upregulated in the *rocA* mutant and *emm*3 isolates (with native truncated RocA), indicating that PepO could also be utilized as a marker for identifying RocA-inactivated isolates, including RocA-truncated *emm*3-type isolates.

Do et al. (39) showed that SpeB expression is activated by the pH-sensitive RopB-SIP quorum-sensing pathway. The present study suggested that PepO endopeptidase could degrade SIP to downregulate SpeB expression. In the *covS* mutant, the transcription of *ropB* is repressed by the nonphosphorylated CovR (22). Consequently, the decrease in intracellular SIP and the low level of RopB could result in the downregulation of SpeB in the *covS* mutant. In the *covR* mutant, the expression of *ropB* is derepressed (33, 48). Therefore, during the stationary phase of growth, the increase levels of RopB and SIP could compensate for the effect of PepO degradation to activate SpeB expression. These results suggest that the increased PepO expression could contribute to the repression of *speB* in the *covS* mutant and reveal that CovR/CovS could modulate RopB-SIP via controlling PepO expression.

Although the mutation rate is not high, it is generally accepted that CovR/CovS-inactivated mutants are selected by immune stress during infection, and these mutants are capable of invading deep tissues, resisting phagocytic attack, and causing severe disease manifestations (12–14, 34). This study demonstrated that PepO, the endopeptidase which regulates SpeB expression by degrading the quorum-sensing peptide SIP, could be used as a marker for the identification of these invasive GAS isolates. PepO is a cytosolic protein and flow cytometry analysis showed that the anti-PepO antibody cannot detect PepO with whole-cell GAS (Fig. S3). The cytosolic protein is not preferred as the detection target; however, the increased production of the hyaluronic acid capsule in the *covR* and *covS* mutants would mask the surface antigens, which may limit the use of surface antigens as targets for identifying these mutants. In conclusion, monitoring the emergence of CovR/CovS-inactivated mutants from patients with wound, deep tissue, or blood GAS infection using PepO could be a potential strategy for early detection of high-risk patients to prevent severe manifestations or poor prognoses.

## MATERIALS AND METHODS

### Bacterial strains and culture conditions.

GAS A20 is the *emm*1-type strain and has been described previously (49). Strain AP3 is a mouse-passaged isolate of A20 which has a frameshift deletion in the *covS* gene (33) and was designed as the *covS* mutant in this study. The *rocA* mutant, RocA-truncated mutant, *hasA* mutants, and CovR/CovS-inactivated clinical isolates have been described previously (26, 31, 33). GAS strains were cultured on Trypticase soy agar containing 5% sheep blood or in tryptic soy broth (Becton, Dickinson and Co., Sparks, MD, USA) supplemented with 0.5% yeast extract (TSBY). E. coli DH5α was purchased from Yeastern (Yeastern Biotech Co., Ltd.; Taipei, Taiwan) and was cultured in lysogeny broth (LB) at 37°C with vigorous aeration. SpeB-inducing peptide (MWLLLLFL; purity: 94.469%) and scrambled control peptide (LLFLWLLM; purity: 92.822%) (38) were purchased from Leadgene Biomedical, Inc. (Tainan, Taiwan). These synthetic peptides were suspended in 100% dimethyl sulfoxide (DMSO) to prepare a 10-mM stock solution and stored at −20°C until use. Working solutions were prepared by diluting the stock solution with 25% DMSO. In SIP- and SCRA-supplemented cultures, bacteria were grown to an optical density at 600 nm (OD_600_) of 0.8. Bacterial pellets were collected via centrifugation and resuspended in acidic TSBY broth (pH 6.0) containing different concentrations of SIP and SCRA for 1 h at 37°C. When appropriate, the antibiotics chloramphenicol (25 and 3 μg/mL for E. coli and GAS, respectively) and spectinomycin (100 μg/mL) were used for selection.

### Bacterial colony morphology.

GAS strains were cultured on blood agar plates (Becton, Dickinson and Company; Sparks, MD, USA) at 37°C with 5% CO_2_ supplementation for 24 h. The images were acquired using a Gel Doc XR+ system (Bio-Rad Laboratories, Inc; Hercules, CA, USA).

### DNA and RNA manipulations.

Bacterial genomic DNA extraction, RNA extraction, and reverse transcription were performed as previously described (50). For sequencing of *hasA* and *hasB*, the *hasA* and *hasB* genes were amplified by PCR with the primers PhasA-F and hasB-R-1, and the PCR products were sequenced with the primers hasA-seq-F, hasA-seqR, hasB-seq-F, and hasB-R-1 (Table 3). Real-time PCR was performed in a 20-μL reaction mixture containing 1 μL of cDNA, 0.8 μL of primers (10 μM), and 10 μL of SensiFAST SYBR Lo-ROX pre-mixture (Bioline Ltd; London, United Kingdom) according to the manufacturer’s instructions. Biological replicate experiments were performed using three independent RNA preparations in duplicates. The expression level of *pepO* was normalized to *gyrA* and analyzed using the ΔΔ*CT* method (Roche LightCycler 96 System, Roche Molecular Systems, Inc.; Pleasanton, CA, USA). All values of the control and experimental groups were divided by the mean of the control samples before statistical analysis (51). Primers used for real-time PCR analysis (Table 3) were designed using Primer3 (v0.4.0, http://frodo.wi.mit.edu) according to the MGAS5005 sequence (NCBI accession no. CP000017.2).

**TABLE 3:** Primers used in this study^*a*^

Primer	Use	Sequence (5′–3′)^*b*^	Reference
PhasA-F	PCR	tcagatgaagttgtactccctgaa	This study
hasB-R-1	PCR/sequencing	gctcaatcataccaccaact	This study
hasA-seq-F	Sequencing	atcgaggtccctgtctttca	This study
hasA-seq-R	Sequencing	cgttttgaagtgataaaagaactcc	This study
hasB-seq-F	Sequencing	ggaatggggaacacgtaaaa	This study
pepO-F	qPCR	tgcctttaaagagcgcacag	This study
pepO-R	qPCR	ctaccccacctagatcagcg	This study
Orf-1-F	Construction	gttaaaaggaggcgcctactTAgtggttattgttactatttttg	38
Orf-1-R	Construction	caaaaatagtaacaataaccacTAagtaggcgcctccttttaac	38
SIP-F-1	Construction	gcgggatccggtcaatagccagatgcgata	38
SIP-R-1	Construction	gcgggatcctcgtgatagggtccacaaca	38
gyrA-F-3	qPCR	cgtcgtttgactggtttgg	33
gyrA-F-3	qPCR	ggcgtgggttagcgtattta	33
PepO-BamHI-F	Construction	gcg ggatcc cgagacatcaatgtcgaaaca	This study
PepO-BamHI-R	Construction	gcg ggatcc gaatctcgtgcaatggttgat	This study
PepO-SacII-F	Construction	tcc ccgcgg taattaggtagttgataaacc	This study
PepO-SacII-R	Construction	tcc ccgcgg cattcgtatctccttatatca	This study
promoter-pepO-F	Construction	gcg ggatcc aacgccatttaggtgaccag	This study
promoter-pepO-R	Construction	gcg ggatcc tgcttttgcacgttttgaag	This study

a qPCR, quantitative PCR.

b The underline indicates the restriction enzyme site.

### Construction of the SIP-inactivated mutants, *pepO* mutants, *pepO trans*-complementary strain, and *rocA trans*-complementary strain.

To inactivate SIP translation, the start codon of the *SIP* open reading frame was replaced with a stop codon (TAG) in the chromosome, according to a previous report (38). The 454-bp PCR product was amplified using the primers SIP-F-1 and SIP-R-1 and ligated into pCN143 through the BamHI site (designated as pCN215). To construct the *pepO* mutants, the *pepO* gene with its upstream (538 bp) and downstream (556 bp) regions was amplified using the primers PepO-BamHI-F and PepO-BamHI-R (Table 3). PCR amplicons were digested with BamHI and ligated into the temperature-sensitive vector pCN143 (33). The *pepO* gene was removed via inverted PCR using the primers PepO-SacII-F and PepO-SacII-R (Table 3) and replaced with the chloramphenicol cassette from Vector 78 (52) to generate pCN210. These plasmids were transformed into GAS strains via electroporation, and the transformants were selected as described previously (33). The deletion of *pepO* and the replacement of TAG in the *SIP* open in the transformants was confirmed via Sanger sequencing. To complement the *pepO* expression, the *pepO* gene with its native promoter (2,553 bp) was amplified using the primers promoter-pepO-F and promoter-pepO-R from the wild-type A20 strain, ligated into the low-copy number vector pTRKL2, and transformed into the *pepO* mutant. The *rocA trans*-complementary strain was constructed as described previously (26).

### Anti-PepO antibody.

The antigenic region of PepO was predicted, and the peptide PDTTYYEEGNEKAEELR and the rabbit anti-PDTTYYEEGNEKAEELR polyclonal antibody were purchased from Leadgene Biomedical, Inc. (Tainan, Taiwan).

### Western blot and Phostag Western blot hybridization.

Bacteria were cultured in TSBY broth to a turbidity of OD_600_ = 1.0. The bacterial culture supernatants were collected, filtrated by a 0.22-μm filter, and used to analyze SpeB and SLO expression. The bacterial cells were disrupted using a bead beater (Mini-Beadbeater, BioSpec Products Inc; Bartlesville, OK, USA). The bacterial cell lysate was centrifuged and total protein in the supernatant was collected for analysis. Total protein was mixed with 6× protein loading dye, boiled for 5 min, and subjected to 10% SDS-PAGE. For Phostag Western blot analysis, the bacterial proteins were mixed with 6× protein loading dye (without boiling) and loaded into a 10% SDS-PAGE containing 10 μM Phostag (Wako Pure Chemical Industries Ltd; Richmond, VA, USA) and 0.5 μM MnCl_2_. The separated proteins were transferred onto polyvinylidene fluoride membranes (Millipore; Billerica, MA, USA). The membranes were blocked with 5% skim milk in PBST buffer (phosphate-buffered saline containing 0.2% vol/vol Tween 20) at 37°C for 1 h. CovR protein was detected using anti-CovR serum (33) and PepO was detected by the anti-PDTTYYEEGNEKAEELR antibody. SpeB and SLO proteins were detected by anti-SpeB (Toxin Technology, Inc; Sarasota, IL, USA) and anti-SLO antibodies (GeneTex; Irvine, CA, USA), respectively. After hybridization, the membrane was washed with PBST buffer and hybridized with a secondary antibody, peroxidase-conjugated goat anti-rabbit IgG (Cell Signaling Technology, Inc; Danvers, MA, USA) at room temperature (25°C to 28°C) for 1 h. The blot was developed using Pierce ECL Western blotting substrate (Thermo Fisher Scientific Inc; Rockford, IL, USA) and the signals were detected using a Gel Doc XR+ system (Bio-Rad Laboratories, Inc; Hercules, CA, USA).

### Statistical analysis.

Statistical analyses were performed using Prism software version 5 (GraphPad Software, Inc; San Diego, CA, USA). Significant differences between multiple groups were determined using analysis of variance (ANOVA). Post-tests for ANOVA were performed using Tukey’s honestly significant difference test. Statistical significance was set at *P < *0.05.
