# Supplementary material for: The Bacterial Markers of Identification of Invasive CovR/CovS-Inactivated Group A Streptococcus
Source: Microbiol Spectr. 2022 Oct 6;10(5):e02033-22. doi: 10.1128/spectrum.02033-22 (PMC9603395; doi:10.1128/spectrum.02033-22)
Supplement: Supplemental file 1 — Tables S1-S2, Fig. S1-S3. Download spectrum.02033-22-s0001.pdf, PDF file, 2.4 MB [file spectrum.02033-22-s0001.pdf]

Supplementary Table S1. The *emm* type, the phosphorylation CovR (CovR~P), and the expression level of SpeB, SLO, and PepO in GAS isolates.

**SPY**	***emm* type**	**CovR~P***	**SpeB#**	**SLO#**	**PepO#**
**301**	1	+	+	+	+
**303**	1	+	+	+	+
**304**	1	+	+	+	+
**143**	1	-	-	++	++
**252**	1	-	-	++	++
**261**	1	-	-	++	++
**306**	12	+	+	+	-
**322**	12	+	+	-	-
**235**	12	-	-	++	++
**201**	22	+	+	-	-
**328**	22	+	+	-	-
**338**	22	+	+	-	-
**363**	22	+	+	+	+
**111**	22	-	-	++	++
**138**	22	-	-	++	++
**173**	22	-	-	++	++
**205**	22	-	-	++	++

**343**	25	-	-	++	++
**213**	44	+	+	-	+
**127**	44	-	-	++	++
**132**	44	-	-	++	++
**380**	44	-	-	++	++
**331**	49	+	+	+	-
**362**	49	+	+	++	++
**337**	49	-	-	++	++
**348**	49	-	-	++	++
**139**	73	+	+	+	-
**141**	73	+	+	+	-
**369**	73	-	-	++	++
**315**	78	-	-	++	++
**177**	81	+	+	-	-
**179**	81	+	+	-	-
**163**	81	-	-	++	++
**267**	87	+	+	+	-
**309**	87	+	+	-	-
**228**	87	-	-	+	++

**136**	89	+	-	+	-
**161**	89	+	+	+	-
**168**	89	+	+	-	-
**178**	89	+	+	-	-
**230**	89	+	+	-	-
**212**	89	-	-	++	++
**215**	90	+	+	++	+
**218**	90	+	+	+	+
**367**	90	-	-	++	++
**373**	90	-	-	+	++
**272**	92	+	-	+	-
**273**	92	+	+	+	+
**276**	92	-	-	++	++
**320**	92	-	-	++	++
**317**	102	+	+	-	+
**319**	102	+	+	-	+
**323**	102	+	+	-	+
**116**	102	-	-	++	++
**122**	102	-	-	+	++

**233**	102	-	-	+	++
**326**	102	-	-	+	++
**340**	113	+	+	-	-
**344**	113	+	+	-	-
**121**	113	-	-	+	++
**365**	113	-	-	+	++

*: +, CovR~P positive; –, CovR~P negative

#: –, negative; +, low-level expression; ++, high level expression

Supplementary Table S2. Nucleotide sequence of *covR/covS*, *rocA*, *hasABC* operon, *speB*, *slo*, and *pepO* in SPY362.

Gene	Nucleotide change	Amino acid change
covR	G108T, A118G	G33V, K40E
covS	A634T	N212I
rocA	A66G	-
ropB	-	-
hasA	C96A, C192A	P33T, P68T
hasB	-	-
hasC	C147T, A539G	D180G
speB	C115A, C378A, C392A, T404A, C406A, C408T, C409A, T410C, T413A	Q39K, D126E, T131N, V135D, P136T, L137T, F138Y
slo	-	-
pepO	-	-

Reference strain: NZ131 (NCBI accession No. CP000829.1)

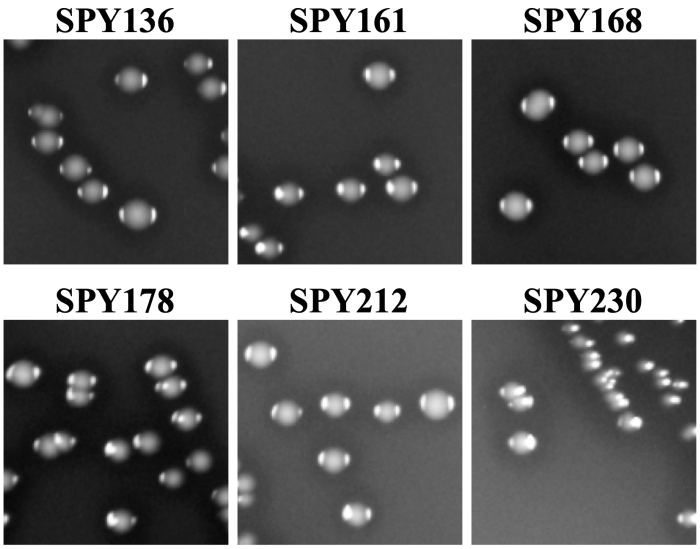

Supplementary Figure S1. The colony morphology of the *emm*89 isolates. The *emm*89 isolates were inoculated on the blood agar plate and incubated at 37ºC with 5% CO_2 for 16 h. The image was recorded by the Gel Doc XR+ system (Bio-Rad).

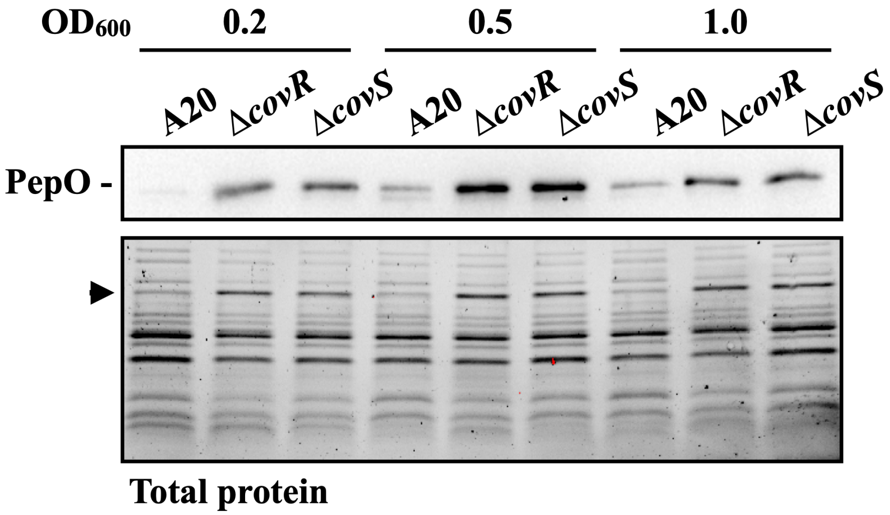

Supplementary Figure S2. The PepO expression in the wild-type A20 strain, the *covR* mutant (Δ*covR*), and *covS* mutant (Δ*covS*) at different growth phases. GAS strains were cultured to the log phase (OD_{600} = 0.2), exponential phase (OD_{600} = 0.5), and stationary phase (OD_{600} = 1.0) and the total proteins were extracted for western blot hybridization. The lower panel of images shows total protein as the internal loading control. An arrow indicates when a signal from only the CovR/CovS-inactivated mutants was identified.

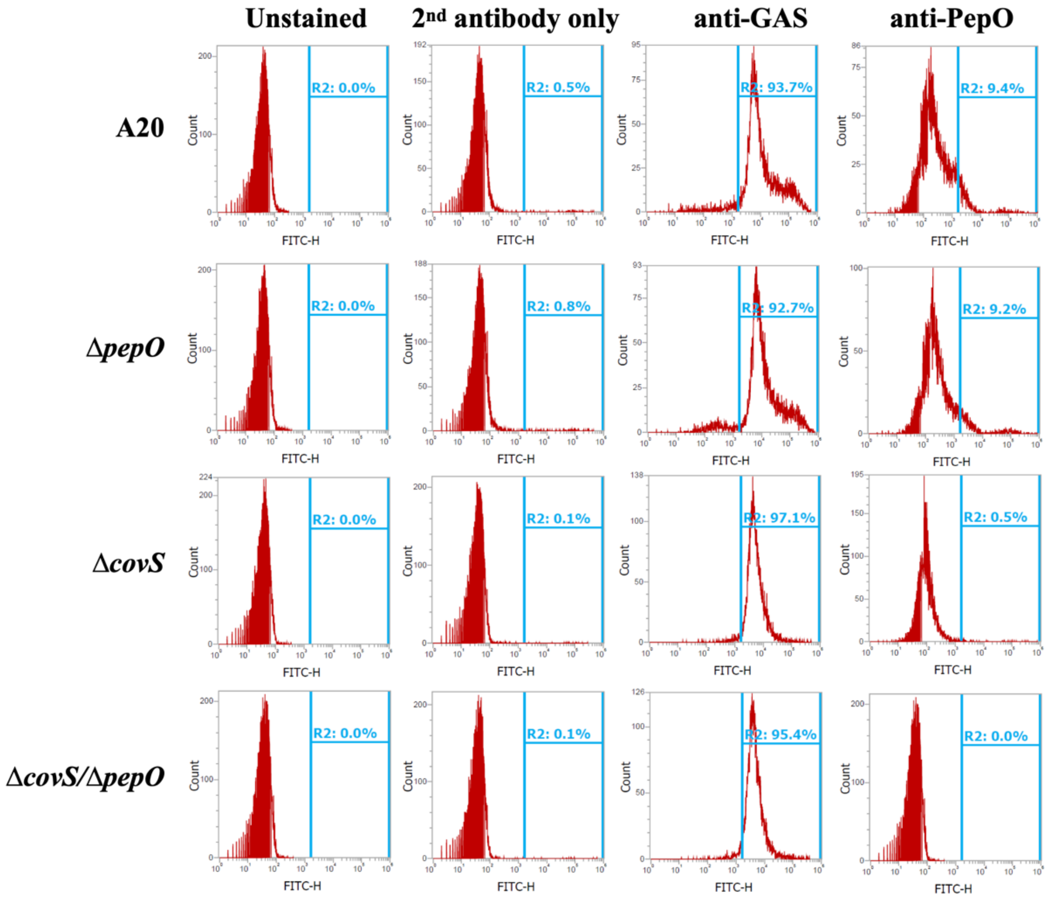

Supplementary Figure S3. Detection of the bacterial surface PepO by using flow cytometry. The exponential phase (OD600 = 0.6) wild-type A20 strain, its *pepO* mutant (Δ*pepO*), the *covS* mutant (Δ*covS*), and the Δ*covS*/Δ*pepO* mutant were collected and fixed by 3% paraformaldehyde at room temperature for 10 min. The fixed bacterial cells were incubated with the primary antibody (anti-GAS antibody 1.25 μg/mL or anti-PepO antibody 15 μg/mL) at room temperature for 1 h. After removing the primary antibodies, the bacterial cells were incubated with the secondary antibody (anti-rabbit IgG-Alexa-488, 1.875 μg/mL) at room temperature for 0.5 h. The signal of PepO on the bacterial surface (total 10,000 bacterial cells) was analyzed by Attune NxT Flow Cytometer (Thermo Fisher Scientific). Unstained and the secondary antibody (2nd antibody only) groups were utilized as the negative controls. Bacteria that incubated with anti-GAS antibody was utilized as the positive control.
